# Supplementary figures and images for: Gene polymorphisms and serum levels of BDNF and CRH in vitiligo patients
Source: PLoS One. 2022 Jul 29;17(7):e0271719. doi: 10.1371/journal.pone.0271719 (PMC9337645; doi:10.1371/journal.pone.0271719)

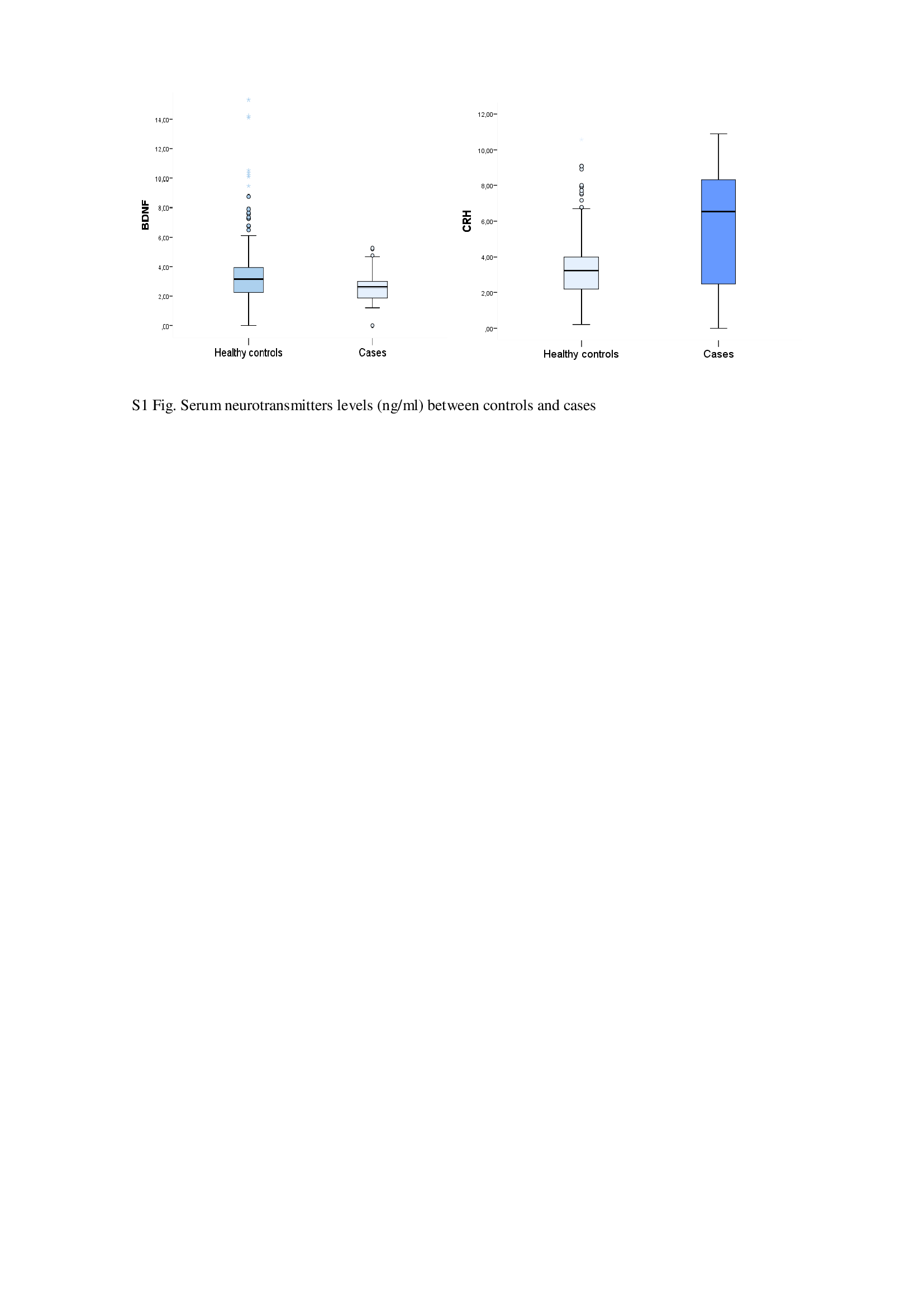

Supplement: S1 Fig — (TIFF) [file pone.0271719.s001.tiff]
