# Supplementary material for: Gene polymorphisms and serum levels of BDNF and CRH in vitiligo patients
Source: PLoS One. 2022 Jul 29;17(7):e0271719. doi: 10.1371/journal.pone.0271719 (PMC9337645; doi:10.1371/journal.pone.0271719)
Supplement: Attachment — Submitted filename: Response to Reviewers PlosOne.docx [file pone.0271719.s004.docx]

# Response to Reviewers’ Comments:

# Journal: PLOS ONE

# Manuscript No:

**Manuscript title:** "Gene polymorphisms and serum levels of BDNF and CRH in vitiligo patients"

**Authors:**	Kussainova et al.

**Journal Requirements:**

When submitting your revision, we need you to address these additional requirements.

1. Please ensure that your manuscript meets PLOS ONE's style requirements, including those for file naming. The PLOS ONE style templates can be found at 
<https://journals.plos.org/plosone/s/file?id=wjVg/PLOSOne_formatting_sample_main_body.pdf> and 
<https://journals.plos.org/plosone/s/file?id=ba62/PLOSOne_formatting_sample_title_authors_affiliations.pdf>

2. We note that the grant information you provided in the ‘Funding Information’ and ‘Financial Disclosure’ sections do not match.

When you resubmit, please ensure that you provide the correct grant numbers for the awards you received for your study in the ‘Funding Information’ section.

Dear Editor,

Thank you for considering our manuscript for publication in PLOS ONE. We did our best to respond to all comments made and to amend the manuscript accordingly.

We have removed the following information from the "Funding" section: ~~This research was carried out as the part of PhD project~~ and added: The author(s) received no specific funding for this work.

3. Thank you for stating the following in the Funding Section of your manuscript:

"This research was carried out as the part of PhD project"

We note that you have provided funding information that is not currently declared in your Funding Statement. However, funding information should not appear in the Acknowledgments section or other areas of your manuscript. We will only publish funding information present in the Funding Statement section of the online submission form. 
Please remove any funding-related text from the manuscript and let us know how you would like to update your Funding Statement. Currently, your Funding Statement reads as follows:

"This research was carried out as the part of PhD project"

Please include your amended statements within your cover letter; we will change the online submission form on your behalf.

Kindly see the changes listed below to evaluate the work done by us.

| **Reviewer 1** | **Changes by the authors** |
| --- | --- |
| Assiya Kussainova and colleagues present an intersting question evalauting the serumm concentrations and polymorphisms in Brain-derived neurotrophic factor (BDNF) and Corticotropin releasing hormone (CRH) in relation to presence or absence of vitiligo. | Thank you very much for reviewing our manuscript and for your thoughtful comments. Your suggestions are very valuable and helped us to improve the quality of our manuscript significantly.  We highlighted all changes made in yellow. |
| **Major comment** | |
| In the introduction psychological or psychiatric disorders are suggested to contribute to the etiology of vitiligo. I would suggest to the authors to rephrase this and stick to the concept psychological disorders may result from vitiligo in accordance with the references given. In the current phrasing the suggestion by the authors is not supported by the references given | Thank you. Done. As proposed, we introduced the following changes:  Nevertheless, vitiligo may serve as a cause for ~~is strongly associated with~~ the wide range of psychological impairments resulting in violation of mental well-being and quality of life [4-6] |
| The authors should add a brief discussion on the neuroedocine inflammatory axis in the introduction to introduce their hypothesis. such as Sternberg EM. J Endocrinol. 2001 | Done. We have added the following information to the "Introduction" section using recommended reference: Corticotropin-releasing hormone (CRH) regulates the hypothalamic-pituitary–adrenal axis (HPA) through activation of its receptor (corticotropin-releasing hormone receptor 1 (CRH-R1)). The CRH gene is also expressed in extracranial tissues, including human skin, and can be synthesized by a variety of immune cells. Human mast cells may contain sufficient amounts of CRH and other stress peptides to mediate its autoimmune and anti-inflammatory effects [16]. Expression of CRH and CRH-R1 mRNA also has been detected in cultured human melanocytes and melanoma cells [17]. Research on vitiligo demonstrated that expression of the CRH-R1 gene is increased in both vitiligo unaffected and vitiligo affected skin. Furthermore, the increased expression of CRH and CRH-R1 correlated with high scores obtained on the Social adaptation assessment scale, which can be explained by the fact that hyperactivation of the cortical-releasing system in skin increases neuroendocrine sensitivity to stress [18]. |
| In the methods no information is provided on the psychological status of the vitiligo patients. | Done. We have entered the following information in the "Materials and Methods” section:  The psychological status of study participants was identified using Generalized Anxiety Disorder-7 (GAD-7) scale. The GAD-7 scores range from 0 to 21, with 0-4 none, 5-9 mild, 10-14 moderate and 15 and more severe levels of anxiety symptoms [25]. |
| In addition information on (auto)immune or inflammatory diseases in the controls is lacking. This should be provided or at least discussed. | Thank you. To overcome this drawback, we added this information to the “Study population section”:  Patients were ineligible for this investigation if they were (i) physically or mentally unable to give informed consent, (ii) had a history of any psychiatric disorder, (iii) had some somatic conditions, such as heart, pulmonary diseases or diabetes mellitus that affected their mental status, (iv) had other skin problems (acne, psoriasis, eczema, etc.)., and (v) presented with autoimmune or inflammatory diseases. |
| The authro should discuss the effects of the polymorphisms studied on serum levels of the mediators as previosuly reported in literature. | Done. We added the following piece of information to the “Discussion” section:  Unfortunately, there is a lack of evidence reporting the effects of the polymorphisms studied on serum levels of the mediators. Two SNP interactions (rs242941 and rs1876828 of the *CRH-R1* gene) were linked to the serum cortisol levels [32].  There is no data on associations of SNPs with serum levels of BDNF in patients with the skin disorders. Few studies described such associations between SNPs of other mediators and their blood levels. For instance, a significant association between IL-6 572 gene polymorphism and its serum level was reported in Egyptian patients with keloid [36]. Another study showed that TT genotype of IL-19 might be a hereditary risk factor for acne vulgaris development, and it was associated with elevated IL-19 serum levels, which could be a marker of acne severity [37]. |
|  | |
| fulll names of the factors measured should be given in the abstract, to make it comprehensible for readers not familiar with the abreviations | Thank you. Done.  The genes encrypting brain-derived neurotrophic factor (BDNF) and corticotropin releasing hormone (CRH) might be the conceivable contributors to the development of vitiligo. …… Genotyping for the rs11030094 polymorphism of the *BDNF* gene and rs242924 of the corticotropin releasing hormone receptor 1 (CRH-R1) gene was performed by real-time polymerase chain reaction (PCR) …… |
| Though written in comprehensible english, the manuscript would benefit if a native english speaker would edit the text. | Done. We didn’t highlight the areas of improvement as in this case the entire manuscript would be highlighted. |
| The discussion should focus more on the potential effects from differences in BDNF and CRH expression levels on the neuroendocrine inflammatory axis, than on the association with neurological or psychiatric diseases. | We addressed the issue of information overload related to the link between neurotransmitters and mental disorders and added the following pieces of information to the “Discussion” section: ~~The variations of BDNF~~ *~~BDNF~~* ~~gene were described in several studies related to the impairment of the essential brain functions. Honea et al. (2013) [28] revealed that~~ ~~BDNF~~ *~~BDNF~~* ~~rs11030094 polymorphism was associated with cognitive decline in the patients with Alzheimer’s disease. Warburton et al. (2016) [17] linked~~ ~~BDNF~~ *~~BDNF~~* ~~rs11030094 SNP to memory dysfunction in patients with newly diagnosed epilepsy. The recent study of Hennings et al. (2019) [20] reported on the association between BDNF~~ *~~BDNF~~* ~~rs11030094 polymorphism and antidepressant response in subjects with major depressive disorder.~~  Our recent study also demonstrated the decreased level of serum BDNF and increased level of serum CRH in vitiligo patients in comparison with the healthy controls [33].  ~~BDNF signaling may be engaged in affective behavior in the form of environmental stresses that cause depression, as well as reduce BDNF mRNA [42]. Based on this, BDNF is extensively investigated in neuropsychiatric diseases such as schizophrenia, severe depressive disorder, MDD, bipolar disorder and various addictions [43]. In the majority of these studies psychiatric disorders were found to be associated with decreased BDNF levels. Moreover, other reports have demonstrated that adequate efficient therapy improves BDNF levels in these patients [44]~~.  Mental distress reduces BDNF level through activation of the hypothalamic-pituitary-adrenal axis. In addition, stress may cause the strain of sympathetic-adrenal-medullary axis, which increases the levels of cortisol and neuroinflammatory cytokines and reduces BDNF levels [52]. |

| **Reviewer 2** | **Changes by the authors** |
| --- | --- |
| 1. Please write the abstract starting with vitiligo and connect with BDNF and CRH,  also please expand the full-form of BDNF and CRF. | Thank you very much for reviewing our manuscript. Your comments helped us to improve its quality.  We highlighted all changes made in yellow    Done. As proposed, we introduced the following changes: Vitiligo is one of the most common hypomelanoses, in which the destruction of functioning melanocytes causes depigmentation of the skin, hair and mucous membranes.  Thank you. Done. The genes encrypting brain-derived neurotrophic factor (BDNF) and corticotropin releasing hormone (CRH) might be the conceivable contributors to the development of vitiligo. |
| 2. Please rewrite the methodology in detail in the abstract. | Done:  The cross-sectional study was carried out between October 2020 and June 2021 in 93 vitiligo patients (age range from 23 to 48 years) and 132 healthy controls (age range from 24 to 52 years). The psychological status of study participants was evaluated using the Generalized Anxiety Disorder-7 (GAD-7) scale. Serum levels of BDNF and CRH were measured with the help of a commercially available sandwich enzyme-linked immunosorbent assay (ELISA) kit. Genotyping for the rs11030094 polymorphism of the *BDNF* gene and for the rs242924 polymorphism of the corticotropin releasing hormone receptor 1 *(CRH-R1)* gene was performed by a real-time polymerase chain reaction (PCR). |
| 3. Conclusion in the abstract is not reflecting the manuscript. Please rephrase it. | Thank you. We rewrote the conclusion as recommended:  ~~These findings may point to a potential role for the aforementioned genetic variations in the development of vitiligo.~~ Our findings demonstrated the association between *CRH-R1* rs242924 and *BDNF* rs11030094 polymorphisms and vitiligo. Further studies need to be carried out in vitiligo patients to confirm the results observed. |
| 4. Please add the latest references in the introduction to describe the vitiligo (<https://www.annsaudimed.net/doi/full/10.5144/0256-4947.2022.96>).  Please cite this reference as it is published in 2022. | Thank you. We have removed previous information from the text and replaced it by the following statement: ~~Vitiligo is a chronic autoimmune skin disease affecting 0.5%-2% of population worldwide [1].~~ Vitiligo is an acquired pigmentry disorder of the skin and mucous membranes which manifests as white macules due to selective loss of melanocytes [1].  Done. ~~1. Bergqvist C, Ezzedine K. Vitiligo: A Review. Dermatology. 2020;236(6):571-592. doi: 10.1159/000506103. Epub 2020 Mar 10. PMID: 32155629.~~  1. Saif GB, Khan IA. Association of genetic variants of the vitamin D receptor gene with vitiligo in a tertiary care center in a Saudi population: a case-control study. Ann Saudi Med. 2022 Mar-Apr;42(2):96-106. doi: 10.5144/0256-4947.2022.96. Epub 2022 Apr 7. PMID: 35380061; PMCID: PMC8982003. |
| 5. Lines no 38/46/54/175/178/181/194/197/198/205/208/226, please update the reference as per the Plos One journal Style format. Replace the year and add the reference number. | Done. |
| 6. Line no 70: Please add controls in the bracket after non-vitiligo | Done:  The non-vitiligo (control) participants were matched by age and sex as the comparator group, and were recruited through community advertisement. |
| 7. Please add the inclusion and exclusion criteria of cases and controls in detail | Thank you. Done:  For the patient to be considered eligible to take part in the study, the following inclusion criteria had to be met: (i) age of 16 years and older, (ii) a verified diagnosis of vitiligo, and (iii) an ability to read and understand Russian or Kazakh. Patients were ineligible for this investigation if they were (i) physically or mentally unable to give informed consent, (ii) had a history of any psychiatric disorder, (iii) had some somatic conditions, such as heart, pulmonary diseases or diabetes mellitus that affected their mental status, (iv) had other skin problems (acne, psoriasis, eczema, etc.)., and (v) presented with autoimmune or inflammatory diseases. |
| 8. Please add the italics form for any name of the gene used in this study? | Thank you very much. Done. |
| 9. Line no 107: What do you mean by 384 line number? | Thank you. We have removed this information from the text: “….and 10 ng of DNA in a total volume of 5 µl. ~~in 384-well plates (Roche, Indianapolis, IN, USA).”~~ |
| 10. Hardy Weinberg Equilibrium, Authors can add the full form and add abbreviated form for using more than 1 time. | Done:  Both case and control groups were in ~~Hardi-Weinberg equilibrium~~ HWE. |
| 11. Authors can add the genetic models in Both tables 2 and 4. | Thank you. Done. **Table 2. Genotype distribution and allele frequencies of ~~CRH-R1~~ *CRH-R1* rs242924** **Alleles** \| **Cases** **N = 93 (%)** \| **Controls** **N = 132 (%)** \| **P** \| **OR (95% CI)**; **G** \| 24 (25.3%) \| 52 (39.4%) \| 0.002 \| 0.52 (0.34-0.79); **T** \| 69 (74.7%) \| 80 (60.6%) \| \| 1.92 (1.27-2.90); **Genotypes** \| \| \| \|; **GG** \| 6 (6.5%) \| 28 (21.2%) \| 0.007 \| 0.26 (0.10-0.65); **GT** \| 35 (37.6%) \| 48 (36.4%) \| \| 1.06 (0.61-1.83); **TT** \| 52 (55.9%) \| 56 (42.4%) \| \| 1.72 (1.01-2.94); **P < 0.05 is significant** **OR – Odds Ratio (95% CI - 95% Confidence Interval)** **A multiplicative model (df=1) was used to identify the association of alleles with the disease** **A general inheritance model (df=2) was used to identify the association of genotypes with the disease** \| \| \| \| **Table 4. Genotype distribution and allele frequencies of ~~BDNF~~ *BDNF* rs11030094** **Alleles** \| **Case** \| **Control** \| **р** \| **OR (95% CI)**; **G** \| 53 (57.5%) \| 61 (46.2%) \| 0.002 \| 1.58 (1.08-2.30); **A** \| 40 (42.5%) \| 71 (53.8%) \| \| 0.63 (0.43-0.93); **Genotypes** \| \| \| \|; **GG** \| 28 (30.1%) \| 33 (25.0%) \| 0.01 \| 1.29 (0.71-2.34); **GA** \| 51 (54.8%) \| 56 (42.4%) \| \| 1.65(0.97-2.81); **AA** \| 14 (15.1%) \| 43 (32.6%) \| \| 0.37(0.19-0.72); **P < 0.05 is significant** **OR – Odds Ratio (95% CI - 95% Confidence Interval)** **A multiplicative model (df=1) was used to identify the association of alleles with the disease** **A general inheritance model (df=2) was used to identify the association of genotypes with the disease** \| \| \| \| |
| 12. Elisa levels can be shown in the figure (Please use R/Python/Any other software) | We have added this information to the Supplement materials:  The serum levels of BDNF and CRH are presented on S1 Fig.    S1 Fig. Serum neurotransmitters levels (ng/ml) between controls and cases |
| 13. Please add about BDNF and CRH and how it is correlated to Vitiligo? | Done. We have added this information to the “Materials and methods” section: The percentage of skin lesions was calculated using the Vitiligo Extent Score (VES) online calculator [24]. The correlation of ELISA levels and VES was not significant (S1 Table). – “Result” section to the Supplement materials: S1 Table. Correlation analysis of BDNF, CRH levels and VES score \| \| BDNF \| CRH; VES score \| r \| -.031 \| -.005; \| p-value \| .769 \| .961 |
| 14. Please add any meta-analysis studies if applicable? | Thank you. We have added the following information to the  "Discussion" section:  Nevertheless, some evidence on association of vitiligo with gene polymorphisms exists. [Tumor necrosis factor (TNF)-α- 308 G/A gene polymorphism](https://pubmed.ncbi.nlm.nih.gov/33370782/) was the most investigated in vitiligo patients. The recent meta-analysis of Giri et al. [38] suggested the involvement of susceptible 'A' allele with vitiligo susceptibility in overall population, whereas the previous studies failed to reveal that TNF-α-308 G/A polymorphism is a genetic risk factor for vitiligo [39, 40]. |
| 15. Please focus on limitations of this study. | Thank you. We have added the following information to the  "Discussion" section:  This study has a number of limitations that have to be discussed. The major limitation comes from its nature of a cross-sectional study, so it was not possible to determine a cause-and-effect relationship between the variables. Second, we utilized the ELISA kit that detected serum BDNF levels without identification of precursor and mature forms of this neuropeptide [14]. In addition, the sample size was rather modest. Still, we believe that our results might be confirmed on samples involving more cases and controls. |
